# COMMUNITY ENGAGEMENT IN OLDER ADULTS WITH COGNITIVE DECLINE: THE USAGE OF INFORMATION COMMUNICATION TECHNOLOGY

**DOI:** 10.1093/geroni/igad104.3236

**Published:** 2023-12-21

**Authors:** Rong-Fang Zhan, Elias Mpofu, Sherif Olanrewaju, Laura Sutherland

**Affiliations:** University of North Texas, Denton, Texas, United States; University of North Texas, Denton, Texas, United States; The Pennsylvania State University, University Park, Pennsylvania, United States; University of North Texas, Denton, Texas, United States

## Abstract

**Background:**

Cognitive decline is typical among older adults, affecting their activities of daily living and community participation. However, the advent of cognitive function support information technologies may improve their community engagement, perhaps more than memory training alone. This study examined information communication technology usage as a mediator of associations of cognitive decline and community engagement in older adults.

**Methods:**

We sampled older adult cases from Round 11 of the National Health and Aging Trends Study data set (N=3817) with cognitive decline in community dwelling (mean age =81.38, SD=6.266), male (n= 1313) and female (n=1771). We utilized multi-level Structural Equation Model (SEM) to examine the mediation of cognitive decline and community connection through the ICTs, as well as direct relationships among memory, orientation, and executive function.

**Results:**

From SEM analysis, an increase of ICTs usage has statistically significantly effects on older adults’ cognitive ability overall (Estimate = 0.103, S.E. = 0.023, C.R. = 4.438, p < 0.001). Cognitive function has a direct impact on the activity orientation function (Estimate=1, p < 0.0011.000). The greater usage of ICTs by older adults is associated with higher levels of community engagement (Estimate = 1.230, S.E. = 0.284, C.R. = 4.338, P < 0.001).

**Conclusion:**

This finding highlights the potential role of ICTs in fostering older adults’ cognitive function for their social interactions and community involvement. Digital age older adults may have greater cognitive function than previous cohort, reducing demand for care services their community participation.

